# Crystal structures of human lysosomal EPDR1 reveal homology with the superfamily of bacterial lipoprotein transporters

**DOI:** 10.1038/s42003-018-0262-9

**Published:** 2019-02-05

**Authors:** Yong Wei, Zi Jian Xiong, Jun Li, Chunxia Zou, Christopher W. Cairo, John S. Klassen, Gilbert G. Privé

**Affiliations:** 1 Princess Margaret Cancer Centre, Toronto, M5G 1L7 ON Canada; 2 Department of Biochemistry, University of Toronto, Toronto, M5S 1A8 ON Canada; 3 Alberta Glycomics Centre and Department of Chemistry, University of Alberta, Edmonton, T6G 2G2 AB Canada; 4 Department of Medical Biophysics, University of Toronto, Toronto, M5G 1L7 ON Canada

## Abstract

EPDR1, a member of the ependymin-related protein family, is a relatively uncharacterized protein found in the lysosomes and secretomes of most vertebrates. Despite having roles in human disease and health, the molecular functions of EPDR1 remain unknown. Here, we present crystal structures of human EPDR1 and reveal that the protein adopts a fold previously seen only in bacterial proteins related to the LolA lipoprotein transporter. EPDR1 forms a homodimer with an overall shape resembling a half-shell with two non-overlapping hydrophobic grooves on the flat side of the hemisphere. EPDR1 can interact with membranes that contain negatively charged lipids, including BMP and GM1, and we suggest that EPDR1 may function as a lysosomal activator protein or a lipid transporter. A phylogenetic analysis reveals that the fold is more widely distributed than previously suspected, with representatives identified in all branches of cellular life.

## Abstract (web summary)

Yong Wei et al. present the crystal structure of the human lysosomal protein EPDR1 and reveal a role in lipid-binding. They show that the larger family of ependymin-related proteins adopt a β-sheet fold previously seen only in bacterial proteins, and that this fold is found throughout the archaea and eukaryotes.

## Introduction

Lysosomes are rich in hydrolases that catabolize proteins, lipids and carbohydrates, but they also contain additional structural and accessory proteins that are required for the normal functioning of the organelle. In addition to their functions in degradation, lysosomes are involved in cell adhesion, cell migration, plasma membrane repair, tumor invasion and apoptosis^1^. The protein composition of lysosomes has been extensively studied over the past several decades, resulting in a fairly consistent parts list for these organelles^2–4^. While most of the known lysosome-associated proteins have at least some degree of functional annotation, a small number of these are identified solely by their localization.

EPDR1 (ependymin-related protein 1) has been identified in several proteomic analyses of mammalian mannose 6-phosphate (M6P) glycoproteins^5–9^, leading to the annotation of EPDR1 as a lysosomal protein of unknown function. M6P glycoproteins are directed to lysosomes via the M6P receptor pathway in the *trans*-Golgi network, but a proportion of these proteins escape this pathway and are secreted from the cell^10^. The lysosomal localization of intracellular EPDR1 was explicitly demonstrated by the subcellular fractionation of mouse brain homogenates^11^. The protein is highly expressed in the brain, but is also present in other tissues^12–14^ and in extracellular fluids^7,9,15–18^. Genetically, EPDR1 has been linked with several diseases^12,13^, including Dupuytren’s disease^19–21^ and primary angle closure glaucoma^22,23^, however these observations do not provide obvious insight into the molecular functions of the protein.

EPDR1 is a member of the ependymin-related (EPDR) family of proteins. The family is named after ependymin, a glycoprotein found in high concentrations within the cerebrospinal fluid of teleost fishes with roles in neuroplasticity, brain and optic nerve regeneration, cold acclimation and behavior^24–32^. Although the true ependymins appear to be restricted to teleost fishes, other branches of the EPDR family have been described that have wider phylogenetic distributions. For example, EPDR1 is a member of the MERP clade that includes representatives from most vertebrates^33^, including sharks, fishes, birds, amphibians and mammals, while more distantly related groups of the family have been identified in tunicates, sea urchins, oysters, sponges and other basal metazoans^13,25,33,34^. Notably, the identification of an ortholog in a choanoflagellate demonstrates that EPDR genes predate the metazoans^35^.

We set out to determine the crystal structure of EPDR1 in order to better understand the molecular functions of this poorly characterized protein. While common sequence-based database searches did not identify any functionally characterized EPDR1 homologs that might provide clues to the role of the protein, more sensitive methods such as HHpred^36^ indicated homology with a family of bacterial proteins related to the LolA lipoprotein transporter^37^ despite pairwise sequence identities below 12%. The crystal structure confirms that EPDR1 adopts the highly curved β-sheet fold that had been previously observed only in bacterial proteins. Structures of EPDR1 with and without an unexpected bound ligand, likely to be a PEG molecule from the crystallization condition, identify an elongated, surface-exposed hydrophobic binding groove in each chain of the homodimer. At acidic pH, EPDR1 can bind to liposomes that contain the anionic lipid bis-monoacylglycero-phosphate (BMP) or the ganglioside GM1, consistent with a role in the degradation or transport of lipids and/or lipoproteins within the lysosome. A phylogenetic analysis shows that the emergence of the EPDR proteins in the vertebrates coincides with the presence of enzymes involved in the biosynthesis and degradation of gangliosides or sulfatides, suggesting a role for this protein in the catabolism of neuronal lipids. We suggest that EPDR1 functions in lipid metabolism and/or transport, possibly with a role as a sphingolipid activator protein, similar to the role of the saposins and the GM2 activating protein^38,39^. Finally, a broad search for more distant members of the LolA/EPDR superfamily reveals that this fold, previously thought to be restricted to the bacteria, is widely distributed throughout the archaea and eukaryotes.

## Results

### Overall structure of EPDR1

**Fig. 1:**
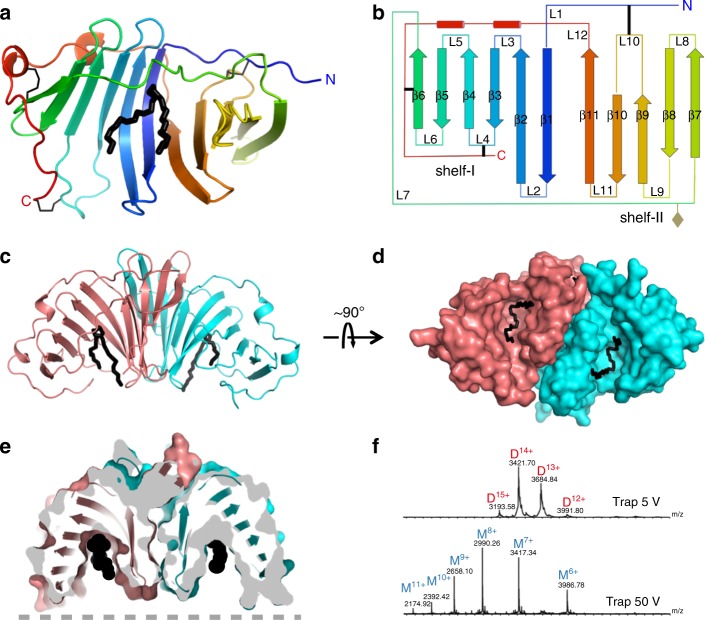
Structure of human EPDR1. **a** Crystal structure of the glycosylated EPDR1 monomer in cartoon representation colored from blue to red, with three disulfide bonds represented as thin black lines. A bound ligand, modeled as a PEG molecule, is shown in thick black lines. **b** Topology of EPDR1 with the secondary structure elements labeled. The glycosylation site is indicated with a grey diamond. **c**, **d** The EPDR1 homodimer in cartoon and surface representations reveals two non-overlapping ligand-occupied grooves. **e** Cut-away view of the dimer in the same orientation as in **c** reveals the two deep ligand-occupied grooves. The black circles indicate the ligand and the dashed grey line represents the presumed position of a lipid bilayer. **f** EPDR1 is dimeric by ESI/MS. Charge states from + 12 to + 15 corresponding to a dimer were observed at low voltage. By increasing the trap collisional energy, the EPDR1 dissociates from a dimer (D, 47889.5 + /- 0.5 Da) to a monomer (M; 23914.1 + /−0.5 Da). The calculated mass of the glycosylated monomer with three intrachain disulfide bonds is 23910.7 Da

**Fig. 2:**
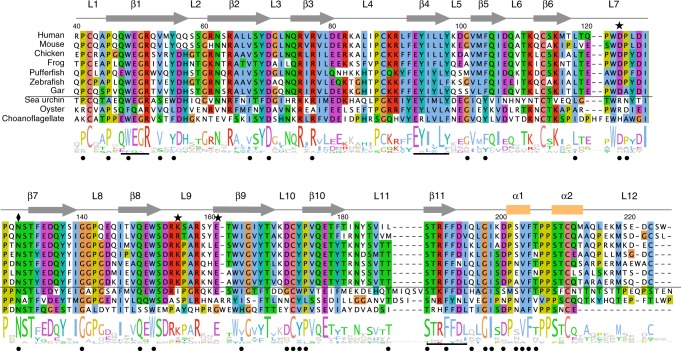
Multiple sequence alignment of EPDR1 orthologs from the MERP subgroup. The secondary structure elements and sequence logo are included above and below the alignment, respectively. Residues are numbered according to the human sequence. Human: *Homo sapiens* NP_060019.2; mouse: *Mus musculus* NP_598826.3; chicken: *Gallus gallus* XP_418830.2; frog: *Xenopus tropicalis* XP_002939463.1; pufferfish: *Takifugu rubripes* XP_003976229.1; zebrafish: *Danio rerio* NP_001002416.1; gar: *Lepisosteus oculatus* XP_006634432.1; sea urchin: *Strongylocentrotus purpuratus* XP_786460.3; oyster: *Crassostrea gigas* XP_011454660.1; choanoflagellate: *Monosiga brevicollis* XP_001750045.1. The glycosylation site at N130 is indicated with a diamond. The thin horizontal line separates vertebrate MERP sequences from the non-vertebrate sequences; vertebrate-specific features include conserved residues D123, K155 and E161 (stars) and the C88-C222 disulfide bond. The underlined motifs are discussed in the text, and the black dots indicate residue positions that are discussed in Fig. 5

We used a piggyBac transposon-based system to overexpress human EPDR1 in HEK293 cells^40^ and purified the overexpressed protein from the culture medium. Crystals were obtained for native and deglycosylated protein. We solved the deglycosylated form by Se-Met SAD phasing and used this structure to solve the glycosylated form by molecular replacement (Table 1). The structure revealed an extended and twisted 11 stranded antiparallel β sheet made up of two smaller sub-sheets consisting of strands 1–6 and strands 7–11, which we refer to as shelf-I and shelf-II, respectively (Fig. 1a, b). The two shelves are linked by antiparallel H-bonding between β1 and β11, placing strands β6 and β7 at opposite ends of the sheet. A long 35 Å loop L7 spanning residues Leu118 to Ser131 connects the outermost strands from the two shelves and packs against the concave surface of the sheet. EPDR1 contains several highly conserved cysteine residues, and their linkage is revealed in the crystal structure. Disulfide-linked residues C42/C172 and C113/C210 are conserved across several EPDR clades, while the C88/C222 linkage is unique to the vertebrate members of the MERP subfamily (Fig. 2)^33,34^. As a result of the positions of these six cysteines, the 9 residues that precede strand β1 are connected to shelf-II by one disulfide bond, while the 33 amino acids that follow strand β11 are connected to shelf-I by two disulfide bonds (Fig. 1b).

**Table 1:** Data collection and refinement statistics

	Glycosylated	Deglycosylated Se–Met
*Data collection*		
Space group	C 2 2 2_1_	C 2 2 2_1_
Cell dimensions		
*a*, *b*, *c* (Å)	102.11, 136.40, 75.91	87.80, 97.48, 189.47
α, β, γ (°)	90, 90, 90	90, 90, 90
Resolution (Å)	42.4–3.1 (3.17–3.11)	61.7–3.0 (3.10–3.00)
*R*_sym_	0.148 (0.896)	0.140 (1.604)
*I* / σ*I*	24.0 (3.4)	15.7 (1.9)
*CC*_*1/2*_	0.967 (0.818)	0.998 (0.714)
Completeness (%)	99.3 (84.3)	99.9 (99.6)
Redundancy	7.0 (6.2)	8.3 (8.4)
*Refinement*		
Resolution (Å)	42.4–3.2	61.7–3.1
No. reflections	9725	17638
*R*_work_ / *R*_free_	0.220 / 0.283	0.255 / 0.278
No. atoms		
Protein	2988	5040
Ligand/ion	56	0
Water	0	0
*B*-factors (Å^2^)		
Protein	86.6	95.9
Ligand/ion	99.6	
R.m.s. deviations		
Bond lengths (Å)	0.011	0.005
Bond angles (°)	1.36	0.88

Values in parentheses are for the highest-resolution shell

The overall shape of EPDR1 resembles a partially opened baseball glove with a deep hydrophobic groove enclosing a volume of approximately 3200 Å^3^ as analyzed by CASTp^41^. The floor of the pocket is lined with mostly hydrophobic residues from strands β1, β2, β3, and β4 from shelf-I, while the rim is formed from loop L7 and the C-terminal loop L12 on one side, and the L2, L9, and L11 hairpins on the other. EPDR1 contains a single glycosylation site at residue Asn130 of loop L7 on the back-side of the glove. As described in more detail below, this fold has been previously observed in the LolA/LolB family of bacterial proteins.

Two EPDR1 chains associate into a tight homodimer through extensive hydrophilic contacts between the convex surfaces of shelf-II (Fig. 1, Supplementary Fig. 1a). This buries approximately 1600 Å^2^ of surface area, and includes major contributions from the L8 hairpin between β7 and β8. The dimerization interface is mostly polar and consists mainly of hydrogen bonds and salt bridges. EPDR1 behaves as a dimer in solution by size exclusion chromatography and a stable homodimer is confirmed by ESI/MS (Fig. 1f).

**Fig. 3:**
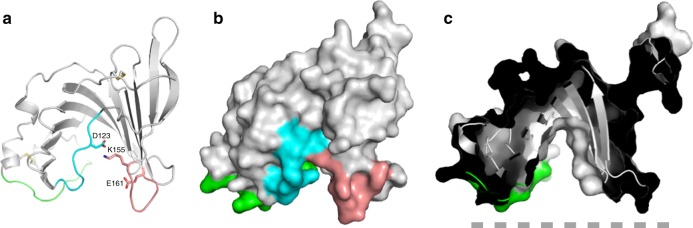
Conserved residues D123, K155 and E161 cluster together at one end of the hydrophobic groove. **a** Cartoon representation of the monomer with loops L7, L9 and L12 colored cyan, pink and green, respectively. Surface representation (**b**) and cut-away view (**c**) of the chain in the same orientation as in **a**. The dashed grey line represents the presumed position of a membrane surface

In the structure of glycosylated EPDR1, the hydrophobic grooves from the two monomers each contain a long continuous tube of electron density, which can be due to a copurifying lipid or a PEG molecule contributed from the crystallization solution (Supplementary Fig. 1b). We were not able to identify copurifying lipids by mass spectroscopy. We modeled this ligand as an extended PEG chain; the U-shaped path of the unidentified ligand follows the floor of the groove and was similar in both protomers. The buried ligand is in van der Waals contact with the hydrophobic side chains of residues M54, L67, Y69, V76, V78, Y94, L96, Y98, M103 on shelf-I, F179, I181, I186, L187, F191 on shelf-II and W122 and L125 in L7 (Fig. 1 and Supplementary Fig. 2). The rim of the groove is rich in charged and polar residues and there is a notable clustering of the conserved, exposed polar side chains D123, K155 and E161 at one end of the groove (Figs 2, 3). As with the C88/C222 disulfide pair, these three amino acids are found only in the vertebrate members of the MERP subfamily and are not present non-vertebrate MERPs, fish ependymins, or other EPDR proteins.

Overall, the shape of the EPDR1 homodimer resembles a hemisphere with an external hydrophilic dome opposite to a flat surface that includes open grooves to two distinct and non-overlapping hydrophobic ligand-binding pockets.

### EPDR1 is a member of the LolA fold superfamily

**Fig. 4:**
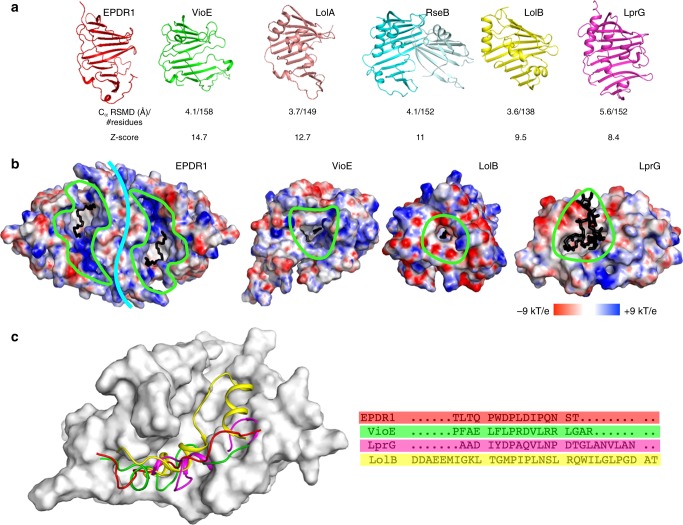
Comparison of EPDR1 to members of the bacterial LolA-type proteins. **a** EPDR1 is structurally similar to VioE (PDB 2ZF4), LolA (1IWL), RseB (2V42), LolB (1IWM) and LprG (3MH9). **b** The surfaces of ligand-occupied EPDR1, VioE, LolB, and LprG are colored by electrostatic potential. The potential for EPDR1 was calculated at pH 4.8, while pH 7 was used for the bacterial proteins. Binding pockets are outlined in green with bound ligands shown as black sticks. EPDR1 is shown as a dimer with the interface marked by a cyan line. **c** The L7 lids from EPDR1, VioE, LprG and LolB shown on the on the surface of EPDR1 along with the sequences

The structure of EPDR1 establishes homology with several proteins from the bacteria, including the lipoprotein localization factors LolA and LolB^37^, the violacein enzyme VioE^42,43^, the sigma-E factor regulatory protein RseB^44,45^ and the lipoprotein LprG^46^ (Fig. 4 and Supplementary Fig. 3). These proteins adopt the LolA/B-type β-clam fold (CATH Superfamily 2.50.20, http://www.cathdb.info/)^47^ despite very low sequence similarity between the proteins. The most notable structural differences between EPDR1 and the two most similar structures, LolA and VioE, include a longer L4 loop that contributes to the shape of the ligand-binding pocket, and a longer L12 C-terminal section that forms part of the rim of the hydrophobic groove. The N- and C-termini in EPDR1 are connected to the core fold by three conserved disulfide bonds, however, none of the known bacterial structures include disulfide bonds. Most of the LolA superfamily structures are monomeric, but exceptions occur, including VioE, which forms a homodimer through a different interface than EPDR1, and RseB, which dimerizes via a separate C-terminal domain. The dimerization interface in EPDR1 appears to be unique to this subfamily and has not been previously observed in other structures of the LolA superfamily.

The proteins from the LolA superfamily carry out wide-range functions. For example, LolA and LolB transport lipoproteins across the periplasm of Gram-negative bacteria for assembly in the outer membrane^48^, and mutations that reduce the hydrophobic character of the LolA pocket result in a loss of transporter activity^49^. A hydrophobic surface is necessary for LprG binding to inner membrane triacylglycerides and transport to outer membrane^46,50^, and RseB binds to the RseA protein to negatively regulate the sigma-E pathway^44,45^. In contrast to the periplasmic location of the preceding proteins, VioE lacks a signal sequence and appears to be a cytosolic enzyme that plays a key role in the biosynthesis of violacein, a purple pigment with antibacterial and cytotoxic properties^42,43^. Despite this functional diversity, a unifying feature of this superfamily is the ability to bind a variety of hydrophobic ligands in the pocket of the highly curved sheet (Fig. 4b). In all cases, the pockets share a hydrophobic cavity with an apolar interior surrounded by a charged rim, but otherwise share little similarity in shape or size. The pocket of EPDR1 is a deep and long groove, while the binding sites in LolA, VioE and LprG are smaller pockets (Figs 1, 3, 4). The sequence and conformation of the L7 lids differ between the members of this fold superfamily, and are responsible, in part, for the diversity in the shape of the ligand-binding pockets (Fig. 4b, c). For example, the lids of LprG and LolB include three short α helices, which function as a flexible gate that controls access to the binding pocket^37,46,48^, while VioE has a shorter, less flexible lid^42,43^. In several cases, the binding pockets are known to remodel to accommodate their ligands, largely through conformational changes in the L7 loops and the C and N termini of the proteins^46,48^. Structures of LolB with bound PEG-MME^37^ and VioE with PEG^43^ illustrate that non-biological ethylene oxide polymers can bind in the hydrophobic pockets of these proteins, consistent with our interpretation of a PEG ligand in our EPDR1 structure. Notably, the ligand-binding groove in EPDR1 is electropositive relative to the bacterial proteins.

**Fig. 5:**
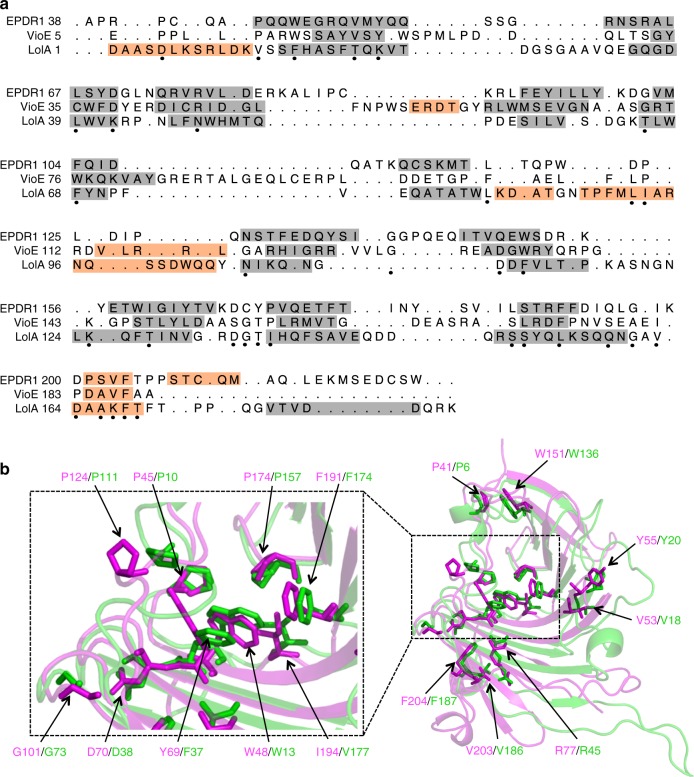
Conservation in the LolA/EPDR superfamily. **a** Structure-based sequence alignment of human EPDR1, *Chromobacterium violaceum* VioE and *E. coli* LolA. Helical segments are shaded in orange and β-strands in gray. Black dots indicate positions where at least two of the three residues are identical, and are equivalent to the sites indicated in Fig. 2. **b** Structural superposition of EPDR1 (magenta) and VioE (green), with conserved side chains shown as sticks. The expanded area on the left highlights the conserved residues in the hydrophobic groove

The structures of EPDR1, VioE and LolA can be superposed with low Cα RSMD values over the 11-stranded β core of the proteins (Fig. 4a), but several of the connecting loops adopt different conformations. A structure-based sequence alignment between EPDR1, VioE and LolA reveals several identical residue pairs that associate in four spatial clusters (Fig. 5). Remarkably, most of the conserved side chains in these clusters adopt similar rotamers leading to consistent spatial orientations. The majority of these residues are hydrophobic, with the exception of D70 and R77 in EPDR1, which align with residues D38 and R45 in VioE, respectively. The largest cluster of residues contributes to the hydrophobic floor of the ligand-binding site (Fig. 5b, left panel).

### Unliganded EPDR1

**Fig. 6:**
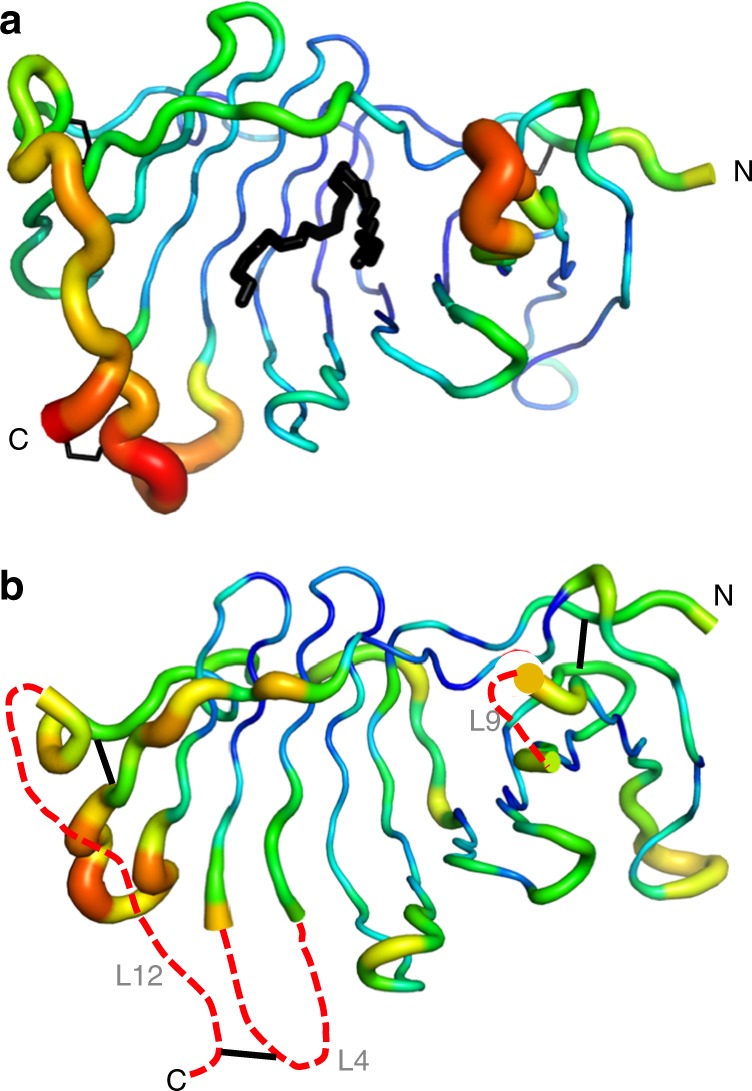
The rim of the lipid-binding pocket includes flexible loops. **a** Glycosylated ligand-bound and **b** deglycosylated apo structures are shown in putty representation in which the thickness and color of the backbone indicate the average crystallographic atomic displacement parameters, colored from low (blue) to high (red). The L4, L9 and L12 sections of the apo structures could not be modeled into the electron density maps and are approximated by red dashed lines

Deglycosylated EPDR1 crystallized in a different packing arrangement with two independent homodimers per asymmetric unit. All three of the crystallographically independent EPDR1 homodimers (one dimer from crystals of the glycosylated protein, and two dimers from crystals of the deglycosylated protein) are similar to each other with Cα RSMDs less than 0.6 Å, indicating little variability within the chains and across the homodimer interface. The main difference between the structures from the two crystal forms is that no ligands were observed within the pockets in any of the deglycosylated structures, and we refer to the deglycosylated structures as apo-EPDR1. In addition to the empty ligand pockets, the loops at the mouth of the groove of apo-EPDR1 had weaker electron density. This is reflected in the higher atomic displacement parameters for loops L6 and L7, while L9, L4 and the C-terminal half of L12 (the latter two are linked by a disulfide bond between residues C88 and C222) could not be traced at all (Fig. 6). These loops make up the flat surface of the half-dome of the homodimer (Figs 1, 3). We suggest that the ridge that lines the opening to the ligand groove is dynamic and flexible in apo-EPDR1 and becomes more ordered upon the binding of a ligand. This effect may be more pronounced with the binding of the natural EPDR1 ligand(s) or through associations with lipid membranes, as described below.

### EPDR1 is detected in lysosomes and in the extracellular medium

**Fig. 7:**
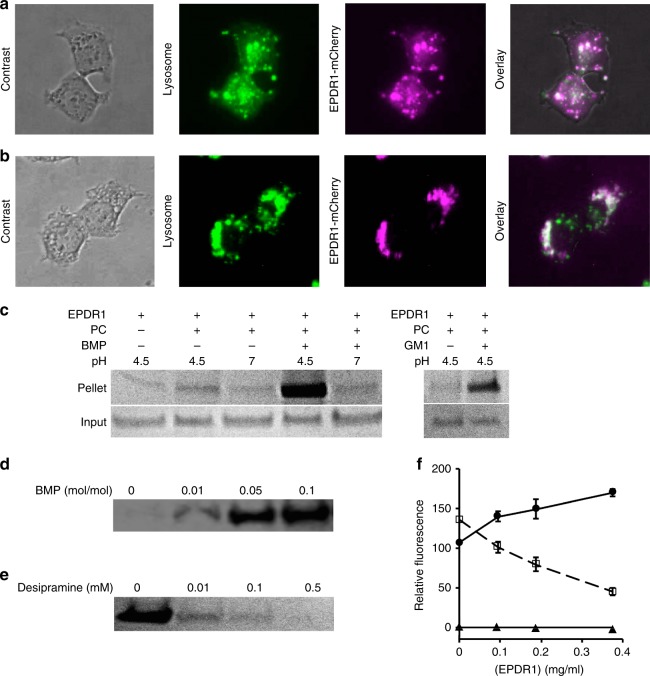
Subcellular localization of EPDR1 and lipid binding. **a** Fluorescence microscopy of HEK293 cells stably transfected with an inducible EPDR1-mCherry fusion construct shows that the fusion co-localizes with a GFP lysosome marker. **b** Exogenously added EPDR1-mCherry fusion protein was taken up by untransfected HEK293 cells and similarly co-localized with a lysosome-tracking GFP marker. **c** EPDR1 associates with liposomes, as determined by a co-sedimentation assay followed by SDS-PAGE and Coomassie blue staining. Binding to liposomes was only observed with the inclusion of the anionic lipid BMP (5% mol/mol) or the ganglioside GM1 (5% mol/mol) at acidic pH. **d** EPDR1 binding increased with the BMP content of the liposomes at pH 4.5, and the cationic amphiphilic drug desipramine reversed the binding at 5% BMP mol/mol levels (**e**). The experiments in **d**, **e** were repeated three times, and representative data are shown. Full size images of the gels are shown in Supplementary Fig. 7. **f** EPDR1 stimulates the neuramidase activity of NEU3 (solid circles), but inhibits NEU4 activity (open squares) on liposomes containing mixed gangliosides. In the absence of neuramindase, EPDR1 did not result in the release of sialic acid (solid triangles). Error bars represent the standard deviation

We expressed full length EPDR1 with a C-terminal mCherry reporter domain in stably transfected HEK293 cells. Live cell imaging showed that the EPDR1-mCherry signal localized mainly to lysosomes (Fig. 7a). In addition to the lysosomal localization, the protein was also detected in the culture medium (Supplementary Fig. 4). We added purified EPDR1-mCherry to the culture medium of untransformed HEK293 cells, and observed uptake and localization to lysosomes (Fig. 7b). The distribution of EPDR1 in both lysosomal and extracellular pools is consistent with previous studies^11^, and the protein has been detected in a variety of fluids, including blood plasma^7^, cerebrospinal fluid^15,16^, urine^9,17^, and seminal fluid^18^. In cultured cells, EPDR1 has been detected in the secretomes of fibroblasts^5^ and adipocytes^51–53^.

### Membrane binding

We performed liposome pull-down assays to characterize the binding of EPDR1 to membranes (Fig. 7c, d). EPDR1 required two of the conditions present in lysosomes for vesicle binding: acidic pH and the presence of anionic lipids. The inclusion of BMP, an anionic lipid that is highly enriched in intralysosomal membranes^39^, or the negatively charged ganglioside GM1 had a similar effect (Fig. 7c). The amount of EPDR1 recovered in the lipidic pellets increased with the concentration of BMP (Fig. 7d). To further verify the role of BMP, we tested the effect of the tricyclic antidepressant desipramine, a cationic amphiphile that partitions into acidic membranes and can neutralize the negatively charged BMP headgroup^54^. Increasing desipramine concentrations reduced the amount of EPDR1 associated with BMP membranes, similar to observations obtained with acid sphingomyelinase^55^. Thus, under acidic conditions where EPDR1 is expected to have a net electropositive charge (Fig. 4b; the protein has a pI of 5.27), EPDR1 can associate with lipid bilayers that contain negatively charged lipids. Notably, while EPDR1 did not have any sialidase activity on ganglioside-containing liposomes, it could stimulate the activity of neuraminidase-3 (NEU3) and inhibit neuramindase-4 (NEU4) (Fig. 7f).

### Phylogenetic and tissue distribution of EPDR1

**Fig. 8:**
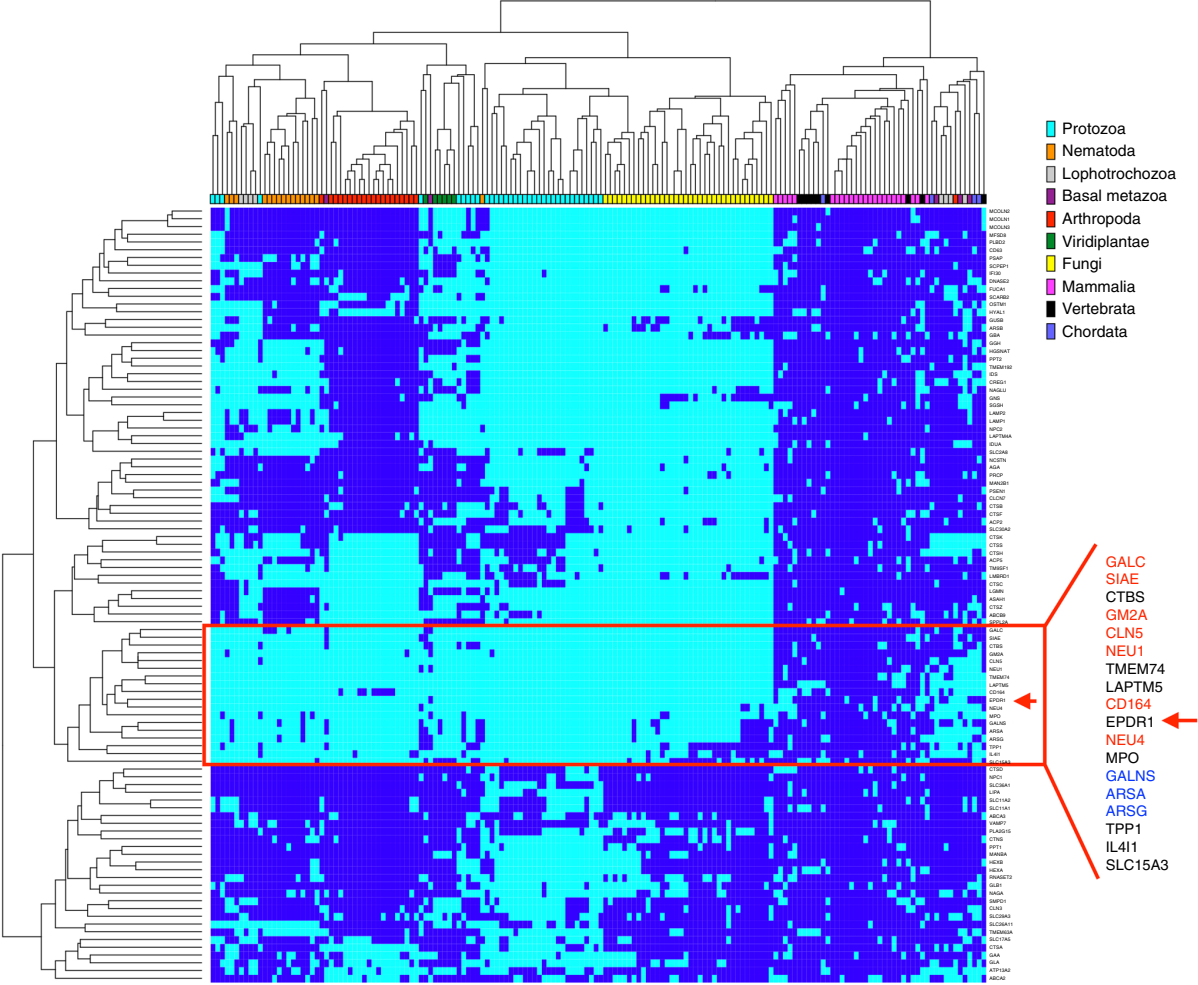
Distribution of orthologs of human lysosomal proteins in the eukaryotes. Hierarchical clustering of orthologs of 100 human lysosomal genes (vertical axis) from 164 species (horizontal axis). The presence of at least one ortholog for a given gene/species pair is indicated with a dark blue node. EPDR1 localizes with a group of genes found predominantly in the vertebrates. The genes in red type are functionally associated with neuronal lipids and/or sialic acid-modified glycolipids/glycoproteins, and the genes in blue type are associated with sulfatase activity

An analysis of the species distribution of orthologs of human lysosomal proteins reveals that EPDR1 clusters with a group of vertebrate and chordate-specific genes (Fig. 8). Notable members of this cluster include proteins involved in the degradation of the gangliosides (NEU1, NEU4 and the GM2 activator protein), as well as the lysosomal sulfatases ARSA, ARSG and GALNS. Gangliosides are sialic acid-containing glycosphingolipids, while sulfatides are galactosphingolipids that contain a sulfate group on the headgroup. In the chordates, these acidic lipids are present at low levels on the surface of most cells, but are highly abundant in nervous tissues^56–58^ and have functional roles in cellular recognition and neurotransmission. The normal homeostasis of these lipids depends on their regulated breakdown in lysosomes, and loss-of-function mutations in the genes required for the catabolism of these lipids are associated with ganglioside and sulfatide lipid storage diseases. Mutations in two of the genes in the EPDR1 cluster, TPP1/CLN2 and CLN5, are found in forms of neuronal ceroid lipofuscinosis^59^. Although the genes that co-occur with EPDR1 were identified by their species distribution, this set also shares overlapping tissue expression patterns in mammals, with particularly high expression levels in the brain and nervous system (https://www.proteinatlas.org, http://www.informatics.jax.org/expression.shtml)^60,61^. At the cellular level, a quantitative study in HeLa cells showed that the intracellular concentration of EPDR1 was similar to that several of the other proteins in the vertebrate cluster from Fig. 8 (CTBS, GM2A, GALNS, ARSA; 10,000–20,000 copies per cell)^62^.

Mice with a homozygous deletion of EPDR1 are viable and have a phenotype involving shortened tibia, hypoactivity, decreased body fat and abnormal behavior as measured by an Open Field test (http://www.mousephenotype.org)^63^. The observation of decreased body fat is notable, as EPDR1 is highly upregulated during adipocyte differentiation^51^ and is selectively secreted by beige^52^ and brown^53^ adipose cells.

Overall, these data suggest that EPDR1 may have a role in the breakdown and/or transport of gangliosides or other acidic glycolipids, possibly as an activator protein. We do not, however, rule out the possibility that EPDR1 may be involved in the catabolism of other macromolecules, including lipoproteins.

### Phylogenetic distribution of LolA/EPDR proteins

**Fig. 9:**
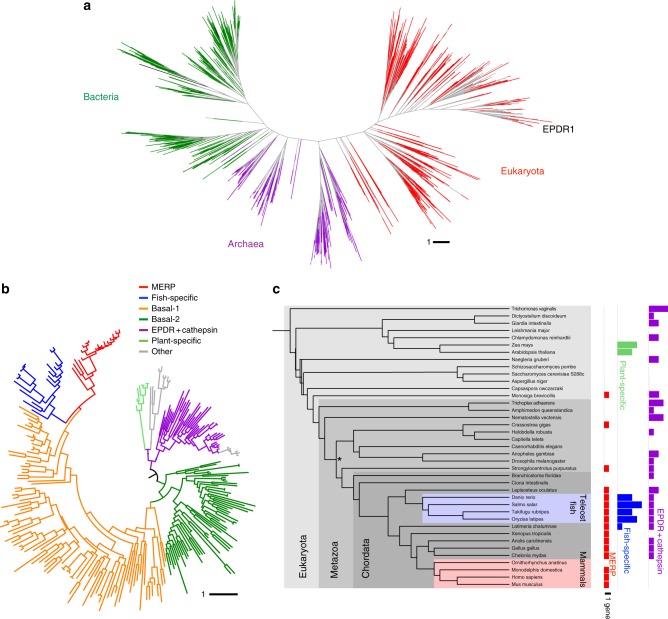
The LolA/EPDR fold is widely distributed in cellular life forms. **a** Unrooted tree of 1782 related sequences from bacteria (green), archaea (purple) and eukaryotes (red). The scale bar indicates the number of substitutions per site. **b** Inferred phylogenetic tree of eukaryotic EPDR proteins based on a multiple sequence alignment of 229 domains from 196 proteins in 40 selected eukaryotic species. Nodes with bootstrap values < 0.8 are collapsed. Major clusters include the EPDR1/EPDR1-like MERP group (red), the fish-specific group (blue, including the true ependymins), two basal/echinoderm groups (orange and dark green), an EPDR + cathepsin group (purple) and a land plant-specific cluster (pale green). **c** Species distribution of selected clades are colored as in **b**. The asterix indicates the protostome/deuterostome branch point

The identification of EPDR1 as a LolA-type protein lead us to search for additional members of this fold superfamily. We used structure-guided sequence alignments and iterative HMMER searches to identify related sequences in the RP15 and RP75 databases of representative proteomes^64^. Manual inspection and reciprocal searches were used to ensure that the collected sequences were all members of the superfamily. Despite the high diversity of the set, the resulting collection of sequences could be aligned with confidence and produced trees with well-defined clusters and reasonable bootstrap values. This analysis revealed multiple clades in each of the bacteria, archaea and eukaryotes, demonstrating that the LolA fold is widely distributed throughout cellular life (Fig. 9a). Given the functional diversity of the characterized bacterial proteins, the functions of the proteins within the archaeal and eukaryotic clades cannot be suggested by orthology.

We identified seven well-defined clades in our clustering of the eukaryotic EPDR proteins (Fig. 9, Supplementary Fig. 5, Supplementary Table 1, and Supplementary Data 1), representing an expansion of earlier analyses of metazoan sequences^33,34^. In nearly all cases, the identified proteins include a signal sequence. Characteristic pairs of conserved cysteine residues mark many of the EPDR subgroups. The most conserved pair, the C113/C210 β6-L12 linkage, is seen in nearly all members of the EPDR family and is one of the most conserved features of the eukaryotic proteins (Supplementary Fig. 5). The presence of conserved signal sequences and disulfide bonds are strong support for endosomal/extracellular locations for the EPDR proteins. Several of the other highly conserved sites correlate with bacterial sites identified in the structure-based alignment in Fig. 5, notably positions D70, R77, W151, P174, F191 and F204. The vast majority of the MERP, fish-specific, and land plant-specific proteins consist entirely of the β-sheet fold and do not include additional C or N terminal extensions, while the EPDR + cathepsin proteins all share a conserved architecture of an N-terminal EPDR domain followed by a cathepsin domain. We did not identify any EPDR family members in the fungi or nematodes, but selected subgroups are represented in some protostome lineages including the arthropods and the molluscs (Fig. 9c and Supplementary Table 1).

### The MERP clade

This subgroup includes the EPDR1 and EPDR1-like sequences that are found throughout the vertebrates, but examples can also be found in lower metazoan species (Figs 2, 9). Members of this clade are almost always present as a single copy in the genomes in which they are detected (Supplementary Table 1). Remarkably, the choanoflagellate *Monsiga brevicolis*, a close unicellular relative of animals^35^, includes several EPDR family sequences, one of which consistently clusters with the EPDR1 subgroup and shares several characteristic sequence features including the MERP-specific QWEGR, EYIxL and STRFFDL motifs (Fig. 2, Supplementary Fig. 5). These three motifs correspond to strands β1, β4 and β11 in the EPDR1 structure. The *Monosiga* gene suggests that the absence of MERP sequences in many of the non-vertebrate metazoan groups may due to the loss of the ancestral gene. MERP genes are found mainly in the deuterostome lineage, however in the protostomes, EPDR1-like genes are found in molluscs but are absent in nematodes and arthopods. It has been proposed that silalic acids predate the deuterostome/protostome split in the animals^65^; this explains the presence of gangliosides in most of the deuterostomes, absence in the nematodes and arthopods, and presence in some molluscs^66^. Thus, the MERP EPDR1-like genes follow the phylogenetic distribution of sialic acids in the eukaryotes.

### The Fish-specific clade

This group includes the FishBrain (including the true ependymins) and the FishTj proteins defined earlier.^33^ Remarkably, this clade includes an example from lobe-finned coelacanth, representing the oldest known living lineage of the tetrapods, but is absent in the spotted gar, which is a ray-finned fish that diverged from teleost fishes before the teleost genome duplication^67^. Members of the Fish-specific clade lack the C88/C222 disulfide bond of the vertebrate MERP proteins, but are otherwise expected to share a highly similar structure to EPDR1. Ependymin is the major glycoprotein component in cerebrospinal fluid of various orders of teleosts^28^, and the abundance of these proteins in fish CSF suggests a role in transport.

### The Basal-1 clade

Genes in this class have undergone rapid expansion in several species, including the ameoba *Naegleria gruberi* (4 genes), the choanoflagellate *Monosiga brevicollis* (5 genes), the placozoa *Trichoplax adhaerens* (13 genes), the mollusc *Crassostrea gigas* (15 genes), the echinoderm *Strongylocentrotus purpuratus* (5 genes) and the cephalochordate *Branchiostoma floridae* (17 genes), but appear to be absent in the vertebrates (Supplementary Table 1 and Supplementary Dataset 1)^34^. The basal-1 group includes the recently described the "clade 1" and "clade 2" subgroups^68^. The majority of the proteins in the latter subgroup have acquired a subset of these proteins have acquired a group-specific pair of cysteines at positions 43 and 126^68^, and we predict that these residues generate a disulfide bond between loops L1 and L7 (the Cα-Cα distance of the equivalent residues in human EPDR1 is 8 Å). Of the twenty-six EPDR proteins encoded in the genome of the crown-of-thorns starfish, 15 have been identified as chemoattractant exoproteins in the water-borne chemical plumes secreted by this organism^34^. Echinoderms synthesize an enormous variety of atypical gangliosides^56,69^, and it is possible that these proteins exist as soluble carriers for sialic-acid lipids as part of a conspecific chemical communication system.

### The EPDR + cathepsin clade

We identified a previously unrecognized set of proteins consisting of an N-terminal EPDR domain fused to a C-terminal L-type cathepsin domain including a cathepsin propeptide inhibitor region. This is notable because most cathepsins are well-characterized lysosomal proteases. Examples of EPDR + cathepsin proteins are widely distributed and found throughout the eukaryotes, but are absent in the mammals. It is not known whether these proteins, which appear to contain a functional cathepsin domain, localize within lysosomes, but all members of this family appear to have a signal sequence and the highly conserved C113/C210 disulfide bond, consistent with an endosomal and/or extracellular localization. In *Dictyostelium discoideum*, the homolog is a secreted factor known as counting factor associated protein (CfaD) and has roles in repressing cell proliferation^70^. The Basal-2, Plant-specific and Other clades are sister groups of this cluster, but most are single-domain proteins consisting entirely of the EPDR fold, with the exception of six genes identified in *Danio rerio* which appear to include a cathepsin propeptide inhibitor domain. 

## Discussion

The LolA fold is well suited for binding to a wide range of guest compounds, and many, but not all, of the characterized members of the fold family bind to lipids and/or lipoproteins. It is notable, however, that VioE is an enzyme that binds to a non-lipidic substrate, and it remains to be seen whether lipid binding is the predominant function of this fold superfamily, or whether other functional roles have been selected in various subgroups.

The crystal structure human EPDR1, with and without bound ligand, represents the first structure of a eukaryotic LolA-type protein, to our knowledge. Despite sharing a common topology with the bacterial proteins, several features distinguish EPDR1 from previous LolA-like structures, including a long and deep ligand-binding groove, unique N- and C-terminal elements, intrachain disulfide links, glycosylation, and an extensive homodimerization surface. EPDR1 has two non-overlapping lipid-binding grooves on the same flat surface of the homodimer, and we presume that this allows the half dome-shaped protein to bind to membranes with an extensive contact surface for the binding and possible extraction and solubilization of target lipids (Supplementary Figure 6). While the vertebrate-specific D123/K155/E161 cluster of polar, conserved residues (Figs 2, 3) may indicate the active site of an enzyme, these residues are highly exposed, present in flexible loops (Fig. 6), and are not strictly conserved (for example, the residue equivalent to K155 is an arginine in the mouse, Fig. 2). Instead, we suggest that these residues are involved in the recognition of a lipid polar headgroup.

Collectively, several lines of evidence suggest that EPDR1 may have a role in lipid catabolism: it is a lysosomal protein, presumably involved in a degradative process, it adopts the LolA-type fold, which is often involved in lipid binding and transport, it is a soluble protein that can bind to negatively charged liposomes, it is unlikely to be an enzyme or structural protein, it can modulate the activities of NEU3 and NEU4, two lysosomal enzymes that hydrolyze sialic acids from gangliosides, it is highly expressed in neural tissues, and the phylogenetic distribution of EPDR1 correlates with ganglioside and sulfatide biology. Although none of these points provide conclusive evidence for the function of the protein, these findings are consistent with a role as a regulator of ganglioside and/or sulfatide processing, and we suggest that EPDR1 functions as an activator protein or a transporter of neuronal lipids.

The LolA fold has adapted to the binding of diverse ligands, and the characterization of some of these indicates a wide range of functional roles, a theme that has been often repeated in evolution. We show that the fold has a broader phylogenetic distribution than previously suspected and is widely represented in numerous subfamilies in the archaea and eukaryotes. The crystal structures presented here establish EPDR1 as a LolA/EPDR fold protein with roles in lipid binding and possibly lipid transport and catabolism, and provide a basis for the further functional characterizations of this and other members of this superfamily.

## Methods

### EPDR1 expression and purification

Human EPDR1 (MERP-1; UCC1; Uniprot ID Q9UM22) was expressed with the piggyBac transposon-based mammalian cell expression system^40^. A fragment coding for protein residues 38–224 with six C-terminal histidines was subcloned into the PB-T-PAF plasmid, which includes an N-terminal tag consisting of a secretion signal, a protein A tag and a thrombin protease cleavage site and confirmed by DNA sequencing. A mixture of the PB-T-PAF, PB-RB, and PBase plasmids at a ratio of 8:1:1 (1 μg total) was transfected into HEK293 GnT1^−^ cells (ATCC). One day after transfection, the cells were trypsinized and distributed into fresh tissue culture plates. Dual drug selection with 10 μg/mL puromycin and 5 μg/mL blasticidin S in DMEM/F12 medium containing 10% v/v fetal bovine serum (FBS) was started at day 3 and continued until the cells reached confluency. Drug-selected cells were distributed into a Nunc EasyFill Cell Factory System (Thermo Scientific) containing 500 mL DMEM/F12 medium supplemented with 10% v/v FBS. Once cells reached confluency, the medium was replaced with 800 mL DMEM/ F12 medium containing 1 μg/mL doxycycline (Sigma) to induce protein expression.

The medium was harvested 4 days later and was loaded onto a 5 mL Ni^2+^ HisTrap HP column (GE Health). Protein was eluted with buffer containing 250 mM imidazole. Fractions containing the fusion protein were pooled, concentrated, and dialyzed against thrombin buffer (50 mM Tris-HCl, 1 mM EDTA, pH 8.0). Thrombin was then added and incubated at 4 °C overnight, followed by a second round of purification by Ni^2+^ chelate chromatography. EPDR1 was further purified by size exclusion chromatography on a Superdex200 16/60 column in 10 mM Tris–HCl pH 8.0 and 100 mM NaCl. Pooled fractions were concentrated to 5 mg/mL and stored at −80 °C. Selenomethionine-substituted protein was expressed in the stable cells^71^, purified as above, and deglycosylated with EndoH in 100 mM NaCl, 50 mM citrate pH 4.5.

### Crystallization and structure determination

Crystals of glycosylated EPDR1 were grown by vapor diffusion by mixing 1 μL protein solution with 1 μL reservoir buffer, and equilibrating against 0.5 mL of reservoir solution (20% PEG3350 and 0.2 M zinc acetate). Selenomethionine-substituted deglycosylated protein was crystallized as above, but with a reservoir solution of 0.2 M lithium sulfate, 0.1 M sodium acetate pH 4.5, 30% PEG8000 and 4% propanediol.

Crystals of selenomethionine-substituted deglycosylated protein were briefly soaked in well solution supplemented with 15% glucose prior to flash freezing, and an anomalous diffraction dataset was collected with 0.9793 Å wavelength x-rays at 100 K at beamline 08ID-1 at the Canadian Macromolecular Crystallography Facility (CMCF) at the Canadian Light Source (CLS). Diffraction images were integrated with HKL 2000^72^ and scaled and merged with AIMLESS^73^. A total of eleven selenium sites were found using phenix.autosol^74^ and these were used to calculate initial phases. Phase improvement and a partial model for four chains was built with phenix.autosol and phenix.autobuild^75^. The model was then iteratively rebuilt with Coot^76^ and refined with phenix.refine^77^ and the PDB_REDO server^78^. The final refined structure contained 7/613 (1%) Ramachandran outliers. For the native glycoprotein, crystals were flash frozen with a 20% glycerol cryo-protectant and diffraction data were collected with 0.6300 Å wavelength x-rays at beamline A1 at Cornell High Energy Synchrotron Source (CHESS). The structure was solved by Phenix molecular replacement using the deglycosylated model. Refinement was performed as for the deglycosylated form. The final refined structure contained 4/361 (1%) Ramachandran outliers. Figures and structural alignments were produced with PyMOL (Schrödinger, LLC). Software used in this project was curated by SBGrid^79^.

### EPDR1 localization and living cell imaging

A fusion protein consisting of full length EPDR1 with its native signal peptide and a C-terminal mCherry fluorescent tag was expressed with the piggyBac transposon-based mammalian cell expression system. Stable cell lines were generated by dual drug selection with 10 μg/mL puromycin and 5 μg/mL blasticidin S and seeded at a density of 10^3^ cells in a 10 cm plate. After 24 h, 1 μg/mL doxycycline (Sigma) and the CellLight lysosomes-GFP reagent (ThermoFisher), which expresses GFP fused to the targeting sequence from Lamp1 (lysosome-associated membrane protein 1), were added to the medium. Cells were imaged on EVOS FL cell imaging system (ThermoFisher) after overnight induction. For the EPDR1 uptake experiments, conditioned media from cells expressing EPDR1-mCherry was collected and the protein was purified by ion exchange chromatography. Non-transfected HEK293 cells were plated at a density of 10^3^ in 10 cm plates and grown for 24 h. Fresh medium containing purified EPDR1-mCherry was added to the plates followed by the addition of the CellLight lysosomes-GFP tracker. After overnight incubation, the medium was removed and cells were washed three times with PBS buffer prior to imaging.

### Liposome binding

Liposomes were prepared by mixing egg PC with BMP or GM1 (Avanti Polar Lipids) in chloroform and solvent was evaporated under N_2_ gas. The lipid film was dispersed by vortex mixing in 5 mM Tris–HCl, pH 7.0. The suspension was subjected to 10 freeze/thaw cycles and sonicated in a bath sonicator for 30 min. Liposomes were diluted to a final concentration of 200 μM total lipid in neutral buffer (50 mM MES, 150 mM NaCl, pH 7) or acidic buffer (50 mM sodium acetate, 150 mM NaCl, pH 4.5). EPDR1 was added to final concentration of 1 μM and incubated for 5 min with gentle mixing. The liposomes were pelleted by centrifugation at 21,000 × *g* for 30 min at room temperature and dissolved in sample buffer for SDS-PAGE.

### Neuramidase activity

An assay to measure the release of sialic acid from gangliosides was adapted from previously described methods^80,81^. Liposomes containing 10% BMP and 10% (mol/mol) bovine brain gangliosides (Millipore-Sigma) were diluted to 5 mM in 100 mM sodium acetate, 150 mM NaCl, pH 4.5 and incubated with various amounts of EPDR1 in the presence of 0.015 units of NEU3, 0.024 units of NEU4, or no enzyme for 1 h at 37 °C. The enzymes were prepared as previously described^81^. The reactions were stopped with the addition of 8 volumes of 200 mM sodium borate pH 10.6, 0.2% w/v malononitrile and heated to 80 °C for 5 minutes. Fluorescence was measured at room temperature with excitation at 375 nm and emission at 434 nm.

### Phylogenomic analysis

A collection of human lysosomal protein sequences^4^ was used to identify orthologs from the genomes of 164 reference species with PhyloPro^82^ and clustered with the heatmap and hclust functions in R. Structure-guided multiple sequence alignments of available LolA-like crystal structures were augmented with additional high-confidence homolog sequences, and these manually curated alignments were used to initiate iterative jackhmmer searches of the RP15 and RP75 databases of representative proteomes (https://www.ebi.ac.uk/Tools/hmmer/search/jackhmmer). Manual inspection and reciprocal searches were used to ensure that the collected sequences were all members of the fold superfamily. PASTA^83^ was used for the refinement of the alignments and generation of maximum likelihood trees with RAxML^84^ (GTR-GAMMA model). Alignment figures were prepared with Jalview^85^ and trees were drawn with with iTOL^86^.

### Mass spectrometry

Mass spectrometry measurements were performed on a Synapt G2S quadrupole-ion mobility separation-time-of-flight (Q-IMS-TOF) mass spectrometer (Waters, Manchester, UK) equipped with a nanoflow ESI (nanoESI) source. Charge states from +12 to+15 were observed for dimer in positive mode. To dissociate EPDR1 dimers, the collisional energy in the trap was increased from 5 V to 50 V.

## Supplementary information

Supplementary Information

Supplementary Data 1

Description of Additional Supplementary Files

## Data Availability

The atomic coordinates and structure factors for glycosylated and deglycosylated EPDR1 have been deposited in the Protein Data Bank (PDB) under the accession codes 6E8N and 6E7O, respectively.
