# Supplementary material for: Crystal structures of human lysosomal EPDR1 reveal homology with the superfamily of bacterial lipoprotein transporters
Source: Commun Biol. 2019 Feb 5;2:52. doi: 10.1038/s42003-018-0262-9 (PMC6363788; doi:10.1038/s42003-018-0262-9)
Supplement: Supplementary file 1 — Supplementary Information [file 42003_2018_262_MOESM1_ESM.pdf]

a

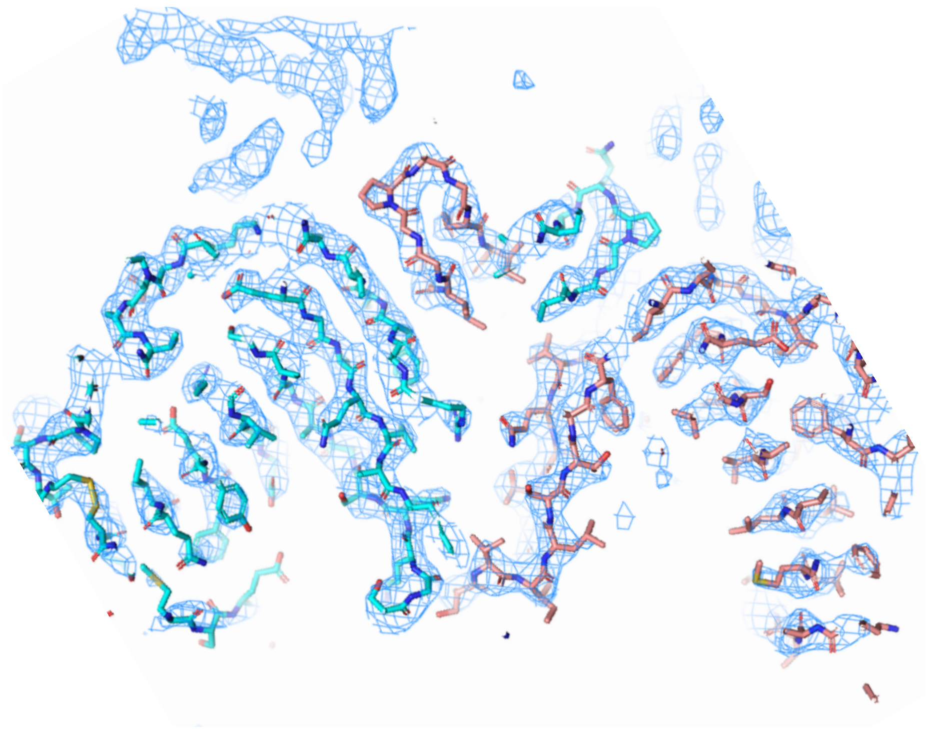

b

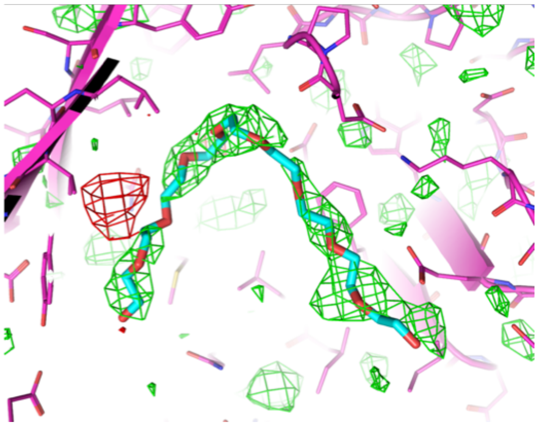

Supplementary Figure 1. **a**. 2Fo-Fc electron density map near the dimer interface of glycosylated EPDR1. The carbons of the two chains are colored blue and pink, respectively. **b.** Difference electron density map following removal of the ligand (modeled as a PEG molecule) and 3 cycles of Phenix torsional angle simulated annealing refinement. The map was contoured at 2.0 sigma. The negative peak to the left (red) is approximately 4 Å from the ligand.

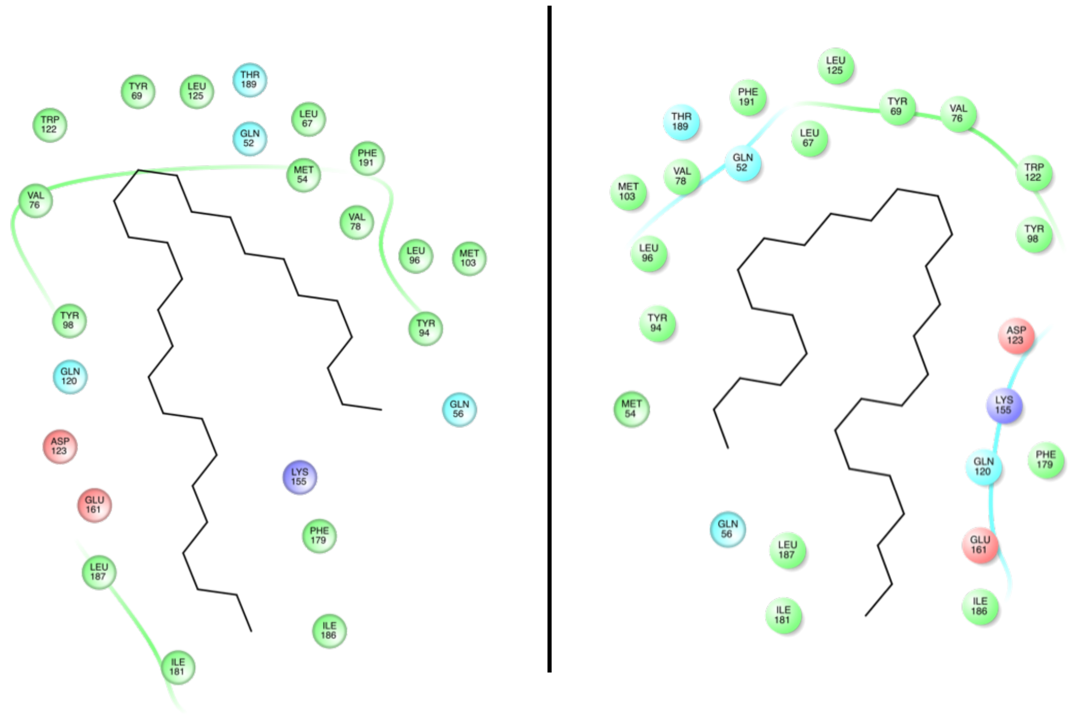

Supplementary Figure 2. Residue contacts of the two ligands in the glycosidated EPDR1 homodimer structure based on a 4 Å cutoff distance. Hydrophobic residues are colored green, polar residues are colored cyan, acidic residues are colored red, and basic residues are colored blue.

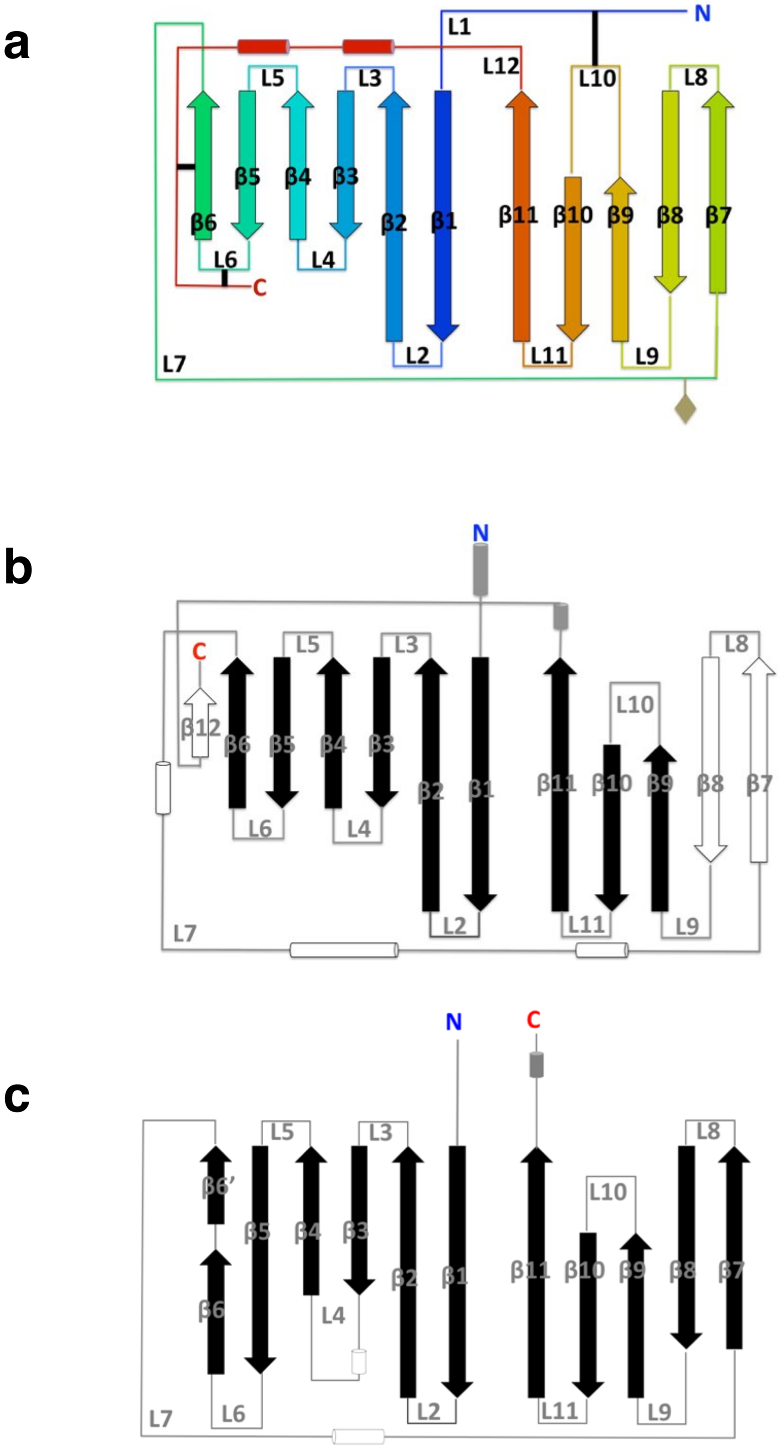

Supplementary Figure 3. Topology diagrams of (**a**) EPDR1, (**b**) LolA (PDB 1IWL) and (**c**) VioE (2ZF4).

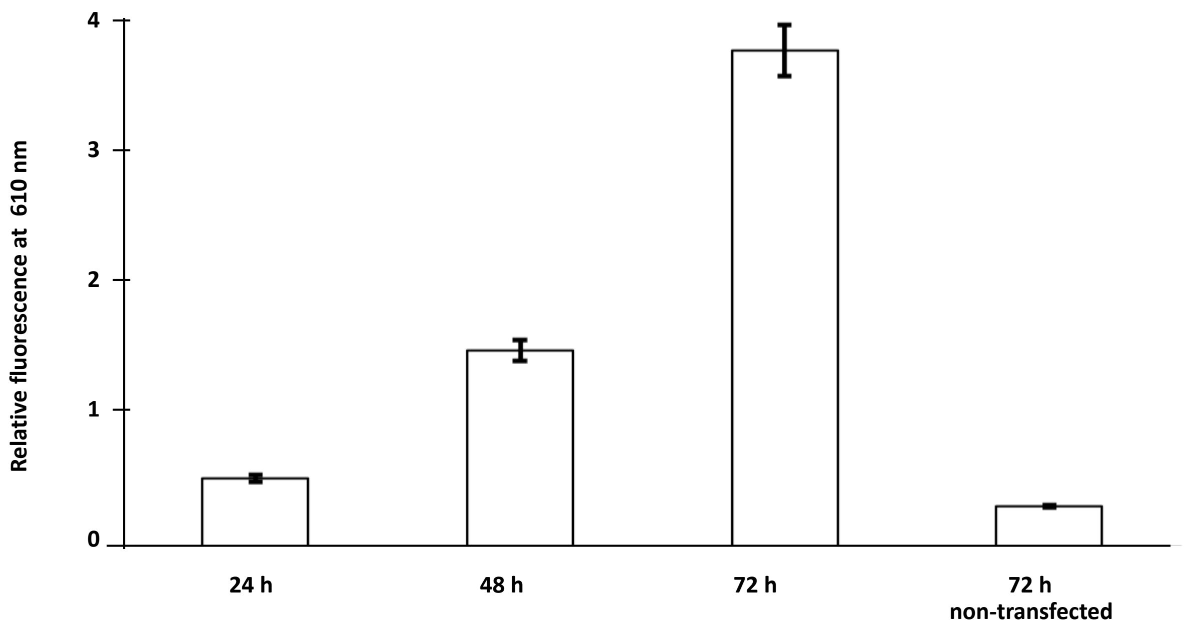

Supplementary Figure 4 Secretion of EPDR1-mCherry fusion protein into the culture medium as measured by fluorescence.

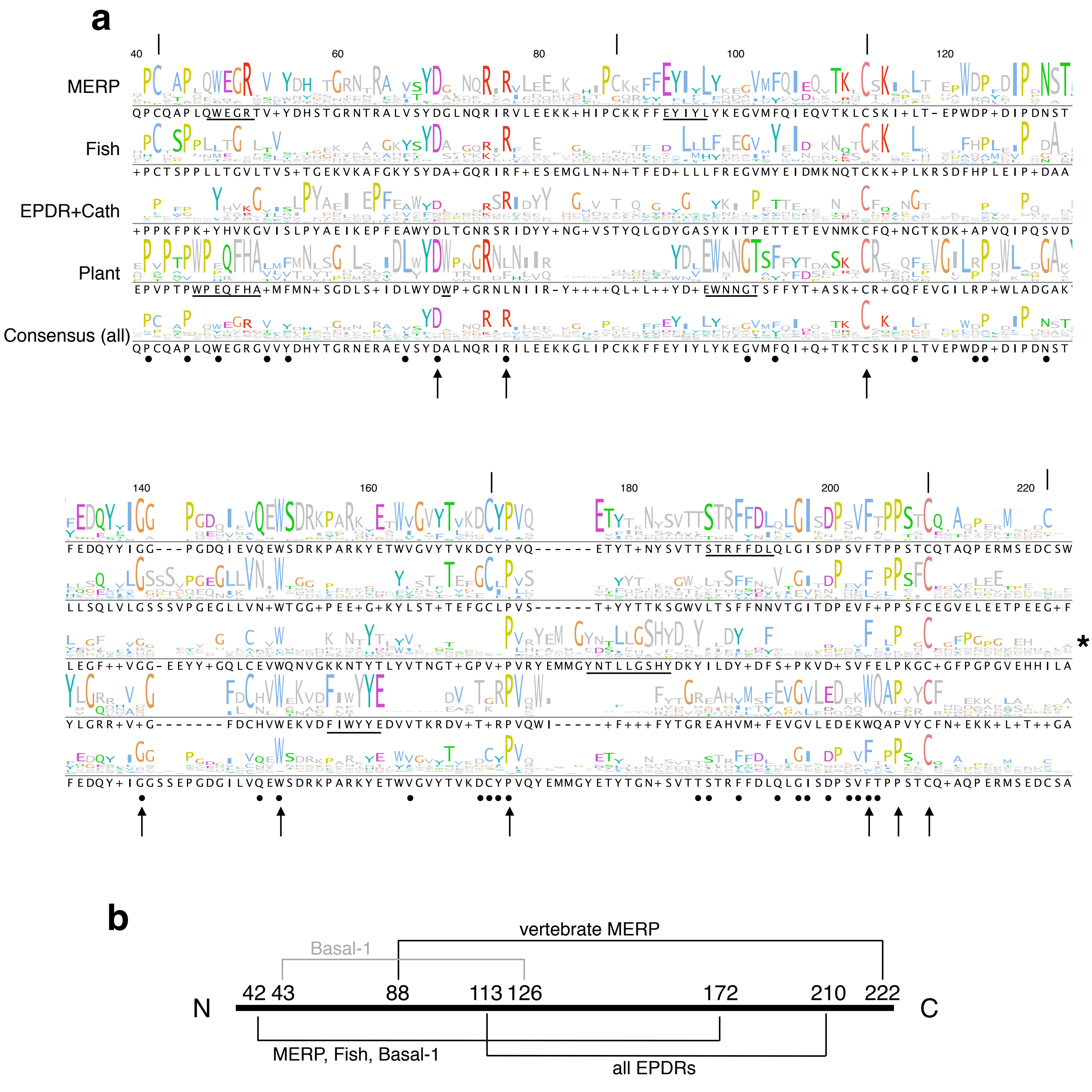

Supplementary Figure 5. **a**. Sequence logos of selected subgroups of the EPDR family (see also Fig. 9 in the main text). Residue numbering is based on human EPDR1. Sequence logos for the basal-1 group can be found in Hall et al., *Nature* **544**, 231-234 (2017). Key cysteine residues are marked with vertical bars above the logos, selected diagnostic sequence motifs are underlined, and sites conserved across the broader family are indicated with upward arrows. Positions that are conserved between the eukaryotic EPDR and bacterial members of the LolA family are marked with black dots as in Figs. 2 and 5 of the main text. The alignments from these clades represent the full length sequences of the predicted mature forms of the proteins following the cleavage of the signal sequences, except for the EPDR+Cath class, which continues into the cathepsin domain (asterix).
b. Disulfide conservation in the eukaryotic EPDRs.

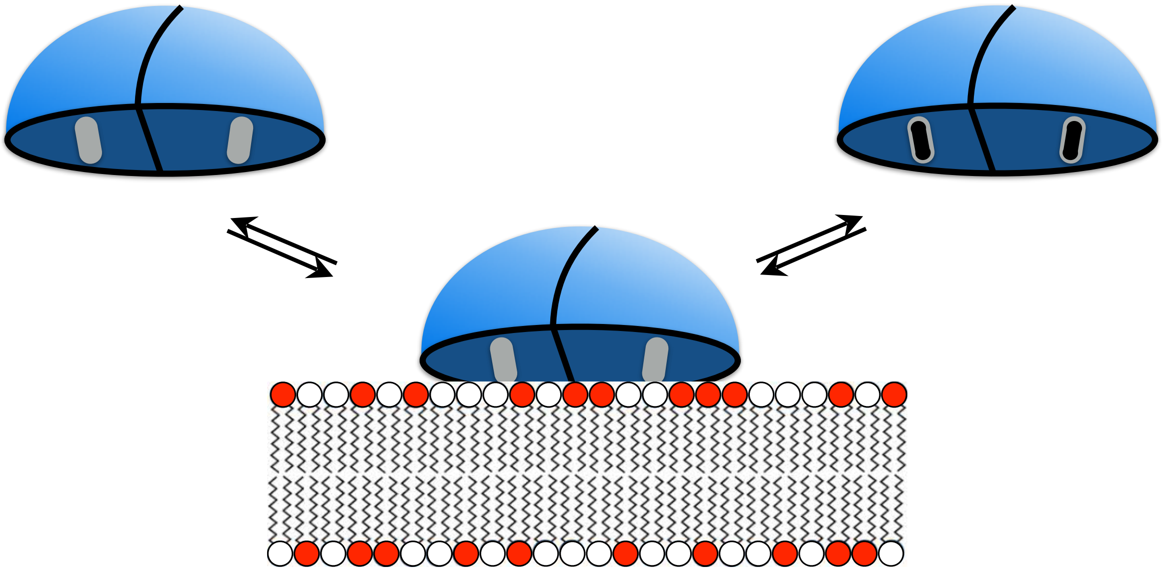

Supplementary Figure 6. Schematic of EPDR1 membrane binding and lipid extraction. The EPDR1 dimer contains two lipid-binding pockets on the flat side of a hemisphere. At acidic pH, the electropositive surface of EPDR1 (blue) interacts with the negatively charged lipids (red headgroups) on membrane surfaces.

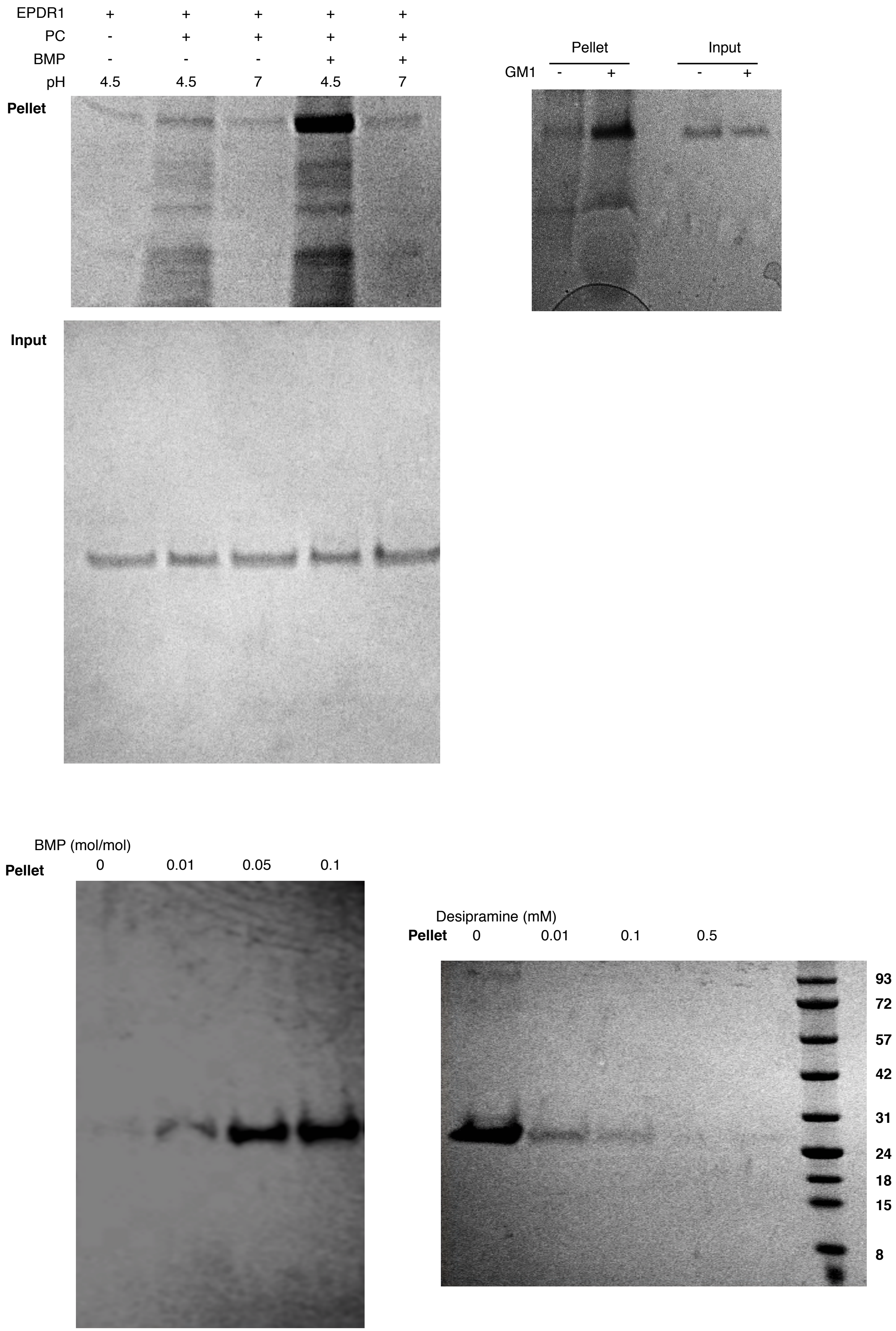

Supplementary Figure 7. Full size images of the Coomassie-stained SDS-PAGE gels shown in Fig. 7 of the main text.

				Subgroup (# of domains)						
species name	common name	# of proteins	# of domains identified	MERP	Fish-specific / Ependymin	Basal-1	Basal-2	EPDR+Cath	Plant-specific	Other
Trichomonas vaginalis		4	4	0	0	0	0	4	0	0
Dictyostelium discoideum	slime mould	2	2	0	0	1	0	1	0	0
Giardia intestinalis		2	2	0	0	0	0	2	0	0
Leishmania major		1	1	0	0	1	0	0	0	0
Chlamydomonas reinhardtii	alga	2	2	0	0	0	0	2	0	0
Zea mays	maize	4	4	0	0	0	0	0	4	0
Arabidopsis thaliana	thale cress	3	3	0	0	0	0	0	3	0
Naegleria gruberi	amoeba	10	10	0	0	4	3	0	0	3
Schizosaccharomyces pombe	**fission yeast**	**0**	**0**	**0**	**0**	**0**	**0**	**0**	**0**	**0**
Saccharomyces cerevisiae	**budding yeast**	**0**	**0**	**0**	**0**	**0**	**0**	**0**	**0**	**0**
Aspergillus niger		**0**	**0**	**0**	**0**	**0**	**0**	**0**	**0**	**0**
Capsaspora owczarzaki		6	7	0	0	1	6	0	0	0
Monosiga brevicollis	Choanoflagellate	7	9	1	0	5	2	1	0	0
Trichoplax adhaerens	Placozoa	16	16	0	0	13	0	3	0	0
Amphimedon queenslandica	sponge	4	6	0	0	0	5	1	0	0
Nematostella vectensis	starlet sea anemone	3	3	0	0	0	1	2	0	0
Lottia gigantea	owl limpet	22	30	1	0	15	13	1	0	0
Crassostrea gigas	Pacific oyster	21	29	1	0	15	13	0	0	0
Helobdella robusta	California leech	1	1	0	0	0	0	1	0	0
Capitella teleta	**bristle worm**	**0**	**0**	**0**	**0**	**0**	**0**	**0**	**0**	**0**
Caenorhabditis elegans	**nematode**	**0**	**0**	**0**	**0**	**0**	**0**	**0**	**0**	**0**
Anopheles gambiae	African malaria mosquito	1	1	0	0	0	0	1	0	0
Drosophila melanogaster	fruit fly	1	1	0	0	0	0	1	0	0
Strongylocentrotus purpuratus	purple sea urchin	10	11	1	0	5	4	1	0	0
Branchiostoma floridae	Florida lancelet (amphioxus)	27	37	0	0	17	13	1	0	6
Ciona intestinalis	vase tunicate, sea squirt	5	6	0	0	4	2	0	0	0
Lepisosteus oculatus	spotted gar	2	2	1	0	0	0	1	0	0
Danio rerio	zebrafish	11	11	1	3	0	0	1	0	6
Salmo salar	Atlantic salmon	7	7	1	5	0	0	1	0	0
Takifugu rubripes	Japanese pufferfish	5	5	1	3	0	0	1	0	0
Oryzias latipes	Japanese medaka	6	6	1	4	0	0	1	0	0
Latimeria chalumnae	coelacanth	3	3	1	1	0	0	1	0	0
Xenopus tropicalis	tropical clawed frog	1	1	1	0	0	0	0	0	0
Anolis carolinensis	green anole	2	2	1	0	0	0	1	0	0
Gallus gallus	chicken	2	2	1	0	0	0	1	0	0
Chelonia mydas	green sea turtle	2	2	1	0	0	0	1	0	0
Ornithorhynchus anatinus	**platypus**	**0**	**0**	**0**	**0**	**0**	**0**	**0**	**0**	**0**
Monodelphis domestica	gray short-tailed opossum	1	1	1	0	0	0	0	0	0
Homo sapiens	human	1	1	1	0	0	0	0	0	0
Mus musculus	house mouse	1	1	1	0	0	0	0	0	0
	domain count:		229	17	16	81	62	31	7	15
	protein count:	196		17	16	75	36	31	7	14

Supplementary Table 1. Distribution of eukaryotic EPDR proteins in selected species. No EPDR proteins were identified in the bold face entries. See also Supplementary Dataset 1.
