# Supplementary material for: Crystal structures of human lysosomal EPDR1 reveal homology with the superfamily of bacterial lipoprotein transporters
Source: Commun Biol. 2019 Feb 5;2:52. doi: 10.1038/s42003-018-0262-9 (PMC6363788; doi:10.1038/s42003-018-0262-9)
Supplement: Supplementary file 3 — Description of Additional Supplementary Files [file 42003_2018_262_MOESM3_ESM.docx]

**Description of Additional Supplementary Files**

**File Name**: Supplementary Data 1

**Description**: Proteins from selected eukaryotes that contain EPDR domains.

List of protein names and sequence identifiers for the proteins used in Fig. 9b,c and Supplementary Table 1.
